# Bracovirus-mediated innexin hemichannel closure in cell disassembly

**DOI:** 10.1016/j.isci.2021.102281

**Published:** 2021-03-08

**Authors:** Chang-Xu Chen, Hao-Juan He, Qiu-Chen Cai, Wei Zhang, Tian-Chao Kou, Xue-Wen Zhang, Shan You, Ya-Bin Chen, Tian Liu, Wei Xiao, Qi-Shun Zhu, Kai-Jun Luo

**Affiliations:** 1 School of Life Sciences, Yunnan University, Kunming 650500, P.R. China; 2 Key Laboratory of the University in Yunnan Province for International Cooperation in Intercellular Communications and Regulations, Yunnan University, Kunming 650500, P.R. China; 3 Biocontrol Engineering Research Centre of Crop Disease & Pest in Yunnan Province, Kunming 650500, P. R. China

**Keywords:** Molecular Biology, Virology

## Abstract

Cell-cell communication is necessary for cellular immune response. Hemichannel closure disrupts communication between intracellular and extracellular environments during polydnavirus-induced immunosuppression in invertebrates. However, the effects of hemichannel closure on cellular immune response are unclear. Here, we examined apoptotic body formation triggered by hemichannel closure in hemocytes of *Spodoptera litura* infected with bracovirus from the parasitic wasp, *Microplitis bicoloratus*. We showed that Microplitis bicoloratus bracovirus (MbBV) induced apoptotic cell disassembly, accompanied by hemichannel closure. Hemocyte apoptotic body formation was caused by the dysregulation of the innexins (Inxs), Inx1, Inx2, Inx3, and Inx4, during the MbBV-mediated inhibition of pI3K/AKT signaling and activation of caspase-3, which cleaved gap junction Inx proteins. Our results showed that hemichannel opening or closure in response to various stimuli, which induces the modulation of Inx levels, could inhibit or activate apoptotic body formation, respectively. Therefore, the “hemichannel open and close” model may regulate the cellular immune response.

## Highlights

• Microplitis bicoloratus bracovirus (MbBV) induced apoptotic cell disassembly
• Apoptotic body formation accompanied innexin hemichannel closure
• MbBV suppressed PI3K/AKT signaling and activated caspase-3
• Innexin cleavage by activated caspase-3 was involved in hemichannel closure

## Graphical abstract

**Figure:**
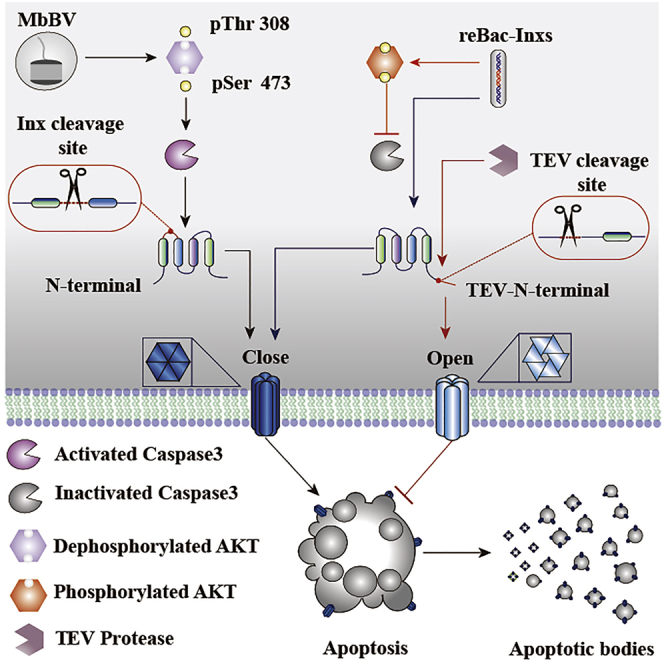

## Introduction

Immunosuppression occurs during parasitization when endoparasitic wasps inject polydnaviruses into their caterpillar hosts (Bitra et al., 2012; Luo and Pang, 2006; Thoetkiattikul et al., 2005). In some host-parasitoid systems, polydnaviruses induce apoptosis in the host hemocytes (Luo and Pang, 2006; Strand and Pech, 1995). Thus far, the fate of apoptotic hemocytes in this process remains unknown.

It is well known that parasitoid polydnaviruses regulate the host's innate immune response. Humoral and cellular immunity are two arms of the insect innate immune systems, and their functions usually overlap (Stanley and Kim, 2014). Humoral immunity mainly mediates the production of antimicrobial peptides, while cellular immunity destroys pathogens through phagocytosis, nodulation, and encapsulation (Ye et al., 2018). For example, hemocytes kill parasitic eggs by forming a multilayer sheath and encapsulating them (Lavine and Strand, 2002; Stanley et al., 2009); during this process, cell-to-cell communication occurs.

The parasitoid polydnavirus is believed to inhibit cell-to-cell communication to protect the parasitic eggs from the immune response of the lepidopteran host. As early as 1995, it was discovered that the Microplitis demolitor bracovirus (MdBV) induced apoptosis and inactivated hemocytes (Strand and Pech, 1995). Follow-up studies also found the same phenomenon in hemocytes infected by the bracoviruses from *Cotesia congregata*, *Microplitis bicoloratus*, and *Snellenius manilae* (Dong et al., 2017; Le et al., 2003; Tang et al., 2021).

Further studies have shown that polydnaviruses use gene products to induce host hemocyte apoptosis, such as the protein tyrosine phosphatase (PTP) of the bracovirus gene family (Ye et al., 2018). PTP can dephosphorylate target proteins, thereby regulating intracellular signal transmission (Eum et al., 2010; Pruijssers and Strand, 2007; Serbielle et al., 2012). The expression of *PTP-H2* (MdBV) in the Sf21 cell line was found to induce apoptosis (Suderman et al., 2008). Therefore, the regulation of *PTP* in host cell signaling pathways may be one of the ways by which polydnaviruses induce hemocyte apoptosis and inhibit host immune function.

Early studies have described the viral ankyrin gene (*vank*), another member of the bracovirus gene family, as an inhibitor of NF-κB; and similar to IκB, it can inhibit the NF-κB signaling pathway of host cells (Bitra et al., 2012; Gueguen et al., 2013). In our latest study, we showed that the Microplitis demolitor bracovirus (MbBV) vank protein interacts with dorsal interaction protein 3 and inhibits the transcription of the translation initiation factor *eIF4E*, thereby inducing the transcription of downstream target genes, such as *inx2* and *inx3* (Cai et al., 2021). This overexpression of Innexin (Inx) 2 and Inx3 promotes apoptosis in Sf9 and Spli221 cells by activating a low level of caspase-3 (Liu et al., 2013).

Inxs are the structural elements of hemichannels. Decrease in the transcription level of *inx* or increase in the number of Inx proteins affect hemichannel function, depending on the steady state levels of Inx on the cell surface (Pang et al., 2015).

Findings from studies on vertebrates have shown that apoptotic cells form apoptotic bodies, which are rapidly cleared by neighboring phagocytic cells to prevent inflammation (Bellone et al., 1997; Brock et al., 2017). However, the mechanism by which polydnavirus-induced apoptosis mediates cell disassembly is not clearly understood. Recently, we found that hemichannel closure involves an N-terminal, elongated Inx hemichannel (Chen et al., 2016; Guo et al., 2015). Cell-cell communication is inhibited upon hemichannel closure as suppressed immune cells cannot initiate encapsulation, nodulation, or phagocytosis. However, the mechanism underlying apoptosis induction due to blocked cell communication and the fate of apoptotic cells has not been determined. We investigated the formation of apoptotic bodies triggered by hemichannel closure following MbBV infection of hemocytes derived from the host, *Spodoptera litura*, or from cell lines derived from *S. frugiperda* pupal tissue.

## Results

### Hemichannel closure and apoptotic cellular disassembly

**Figure 1:**
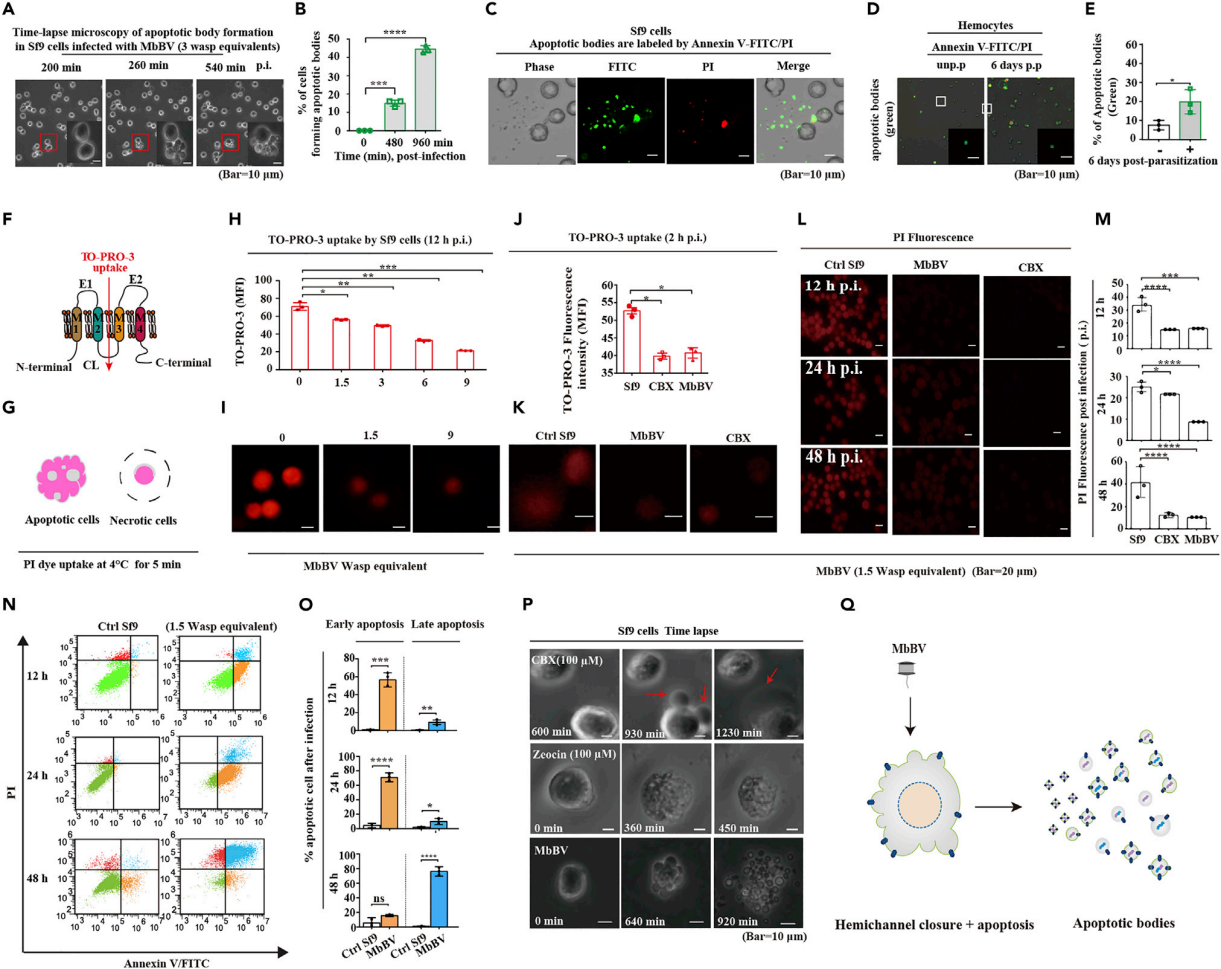
MbBV triggered apoptotic cell hemichannel closure and disassembly (A and B) Time-lapse microscopy of apoptotic body formation triggered by MbBV. The red frame shows cell disassembly. Scale bar, 10 μm; n = 3. ∗∗∗p < 0.001, ∗∗∗∗p < 0.0001, unpaired Student's *t*-test with Holm-Sidak method for multiple *t* test. (C) Annexin V-FITC/PI labeling of apoptotic bodies. Scale bar, 10 μm. (D and E) Microscopy of apoptotic body formation triggered by parasitization by the wasp, *Microplitis bicoloratus*, of the host caterpillar, *Spodoptera litura*, using Annexin V-labeled FITC. The white frame shows apoptotic bodies. Scale bar, 10 μm; n = 3. (F) Schematic of TO-PRO-3 dye uptake from extracellular to intracellular environments through an open hemichannel. (G) Schematic of the difference between PI uptake by apoptotic and necrotic cells at 4°C for 5 min. (H and I) MbBV closed the hemichannels in a viral-dosage-dependent manner using TO-PRO-3 uptake. Scale bar, 20 μm; n = 3. ∗p < 0.05, ∗∗p < 0.01, ∗∗∗p < 0.001, unpaired Student's *t* test with Holm-Sidak method for multiple *t* test. (J and K) TO-PRO-3 uptake of cells infected by MbBV at 2 hr post-infection (p.i.). Scale bar, 20 μm. Unpaired Student's *t*-test with the Holm-Sidak method for multiple *t* test. Scale bar, 20 μm. (L and M) PI uptake of cells infected by MbBV at 12, 24, and 48 hr p.i. Scale bar, 20 μm. ∗p < 0.05, ∗∗∗p < 0.001, ∗∗∗∗p < 0.0001, unpaired Student's *t*-test with Holm-Sidak method for multiple *t* test. (N and O) Flow cytometric detection of apoptotic Sf9 cells infected by MbBV using Annexin V/PI compared to control (Ctrl). Yellow indicates early apoptosis, and blue indicates late apoptosis. ∗p < 0.05, ∗∗p < 0.01, ∗∗∗p < 0.001, ∗∗∗∗p < 0.0001, unpaired Student's *t*-test with Holm-Sidak method for multiple *t* test; n = 3. (P) Time-lapse microscopy of cells treated with CBX, zeocin, and MbBV. Scale bar, 10 μm. (Q) Schematic of apoptotic body formation during hemichannel closure. Cells infected by MbBV showed hemichannel closure and apoptosis and disassembled to form apoptotic bodies. See also Videos S1, S2, and S3.

To investigate the disassembly of MbBV-induced apoptotic cells forming apoptotic bodies, we used hemocytes of the host, *S. litura*, its cell line, Spli221, and *S. frugiperda*-derived Sf9, which can undergo inducible apoptosis caused by MbBV infection (Figure 1). We unexpectedly observed that MbBV induced the disassembly of Sf9 cells *in vitro* during time-lapse microscopy. Using the same quantity of bracovirus for infection (three wasp equivalents) and increasing the incubation period, we observed that more cells formed apoptotic bodies, some of which could be labeled using Annexin V-FITC and some, like late apoptotic cells, using Annexin V-FITC and propidium iodide (PI) (Figures 1A‒1C).

To determine whether this apoptotic induction occurred naturally in the wasp host, *S. litura*, we compared hemocytes from non-parasitized and parasitized hosts 6 days post-parasitization. We found a significantly higher number of apoptotic bodies in the hemocytes from parasitized hosts than in those from non-parasitized hosts (Figures 1D and 1E).

We had previously reported hemichannel closure during reBac-virus infection (Chen et al., 2016; Guo et al., 2015). Hence, to determine whether hemichannel closure occurred during MbBV infection of Sf9 cells, we used TO-PRO-3, which can pass through open hemichannels (Figure 1F), and found that MbBV significantly decreased TO-PRO-3 uptake in a viral-dose-dependent manner (Figures 1H and 1I). Carbenoxolone (CBX) is a pannexin and connexin hemichannel/gap junction inhibitor, which inhibits Sf9 hemichannel opening (Luo and Turnbull, 2011). We determined that both MbBV and CBX inhibited Sf9 hemichannels to similar extents (Figures 1J and 1K).

Based on the information regarding MbBV-induced apoptosis (Luo and Pang, 2006), we tested our hypothesis that hemichannel closure in MbBV-infected cells would persist throughout the apoptotic process triggered by the virus. We performed a set of assays to detect hemichannels in the different stages of apoptosis using PI and Annexin V-labeled FITC in MbBV-infected cells. PI can pass through hemichannels without endocytosis at 4°C for 5 min (Luo and Turnbull, 2011) (Figure 1G). Similar to hemichannel closure by CBX, we observed hemichannel closure during MbBV infection at 12, 24, and 48 hr post-infection (p.i.) (Figures 1L and 1M). Flow cytometric analysis revealed an increase in early apoptotic cells at 12 and 24 hr but not at 48 hr p.i. and a significant increase in late apoptotic cells from 12 to 48 hr p.i. but not 24 hr (Figures 1N and 1O).

Next, we investigated the relationship between hemichannel closure and apoptotic body formation by comparing the effects of CBX, zeocin, an inducer of apoptosis and DNA double-strand breaks (Delacôte et al., 2007), and MbBV. CBX induced membrane blebbing (Figure 1P, Video S1) but did not induce cellular disassembly. Zeocin killed cells but did not induce cellular disassembly (Figure 1P, Video S2). Interestingly, only MbBV induced cell apoptosis and promoted apoptotic cell disassembly (Figure 1P; Video S3). These findings indicate that MbBV induced apoptosis in cells along with the formation of apoptotic bodies, followed by hemichannel closure and the disassembly of cells (Figure 1Q). These results suggest that apoptosis and hemichannel closure are required for apoptotic cell disassembly.

Video S1. CBX induced membrane blebbing, related to Figure 1

Video S2. Zeocin induced cell death but not cellular disassembly, related to Figure 1

Video S3. MbBV induced cell apoptosis and promoted the disassembly of apoptotic cells, related to Figure 1

### MbBV inhibited innexins in cell disassembly

**Figure 2:**
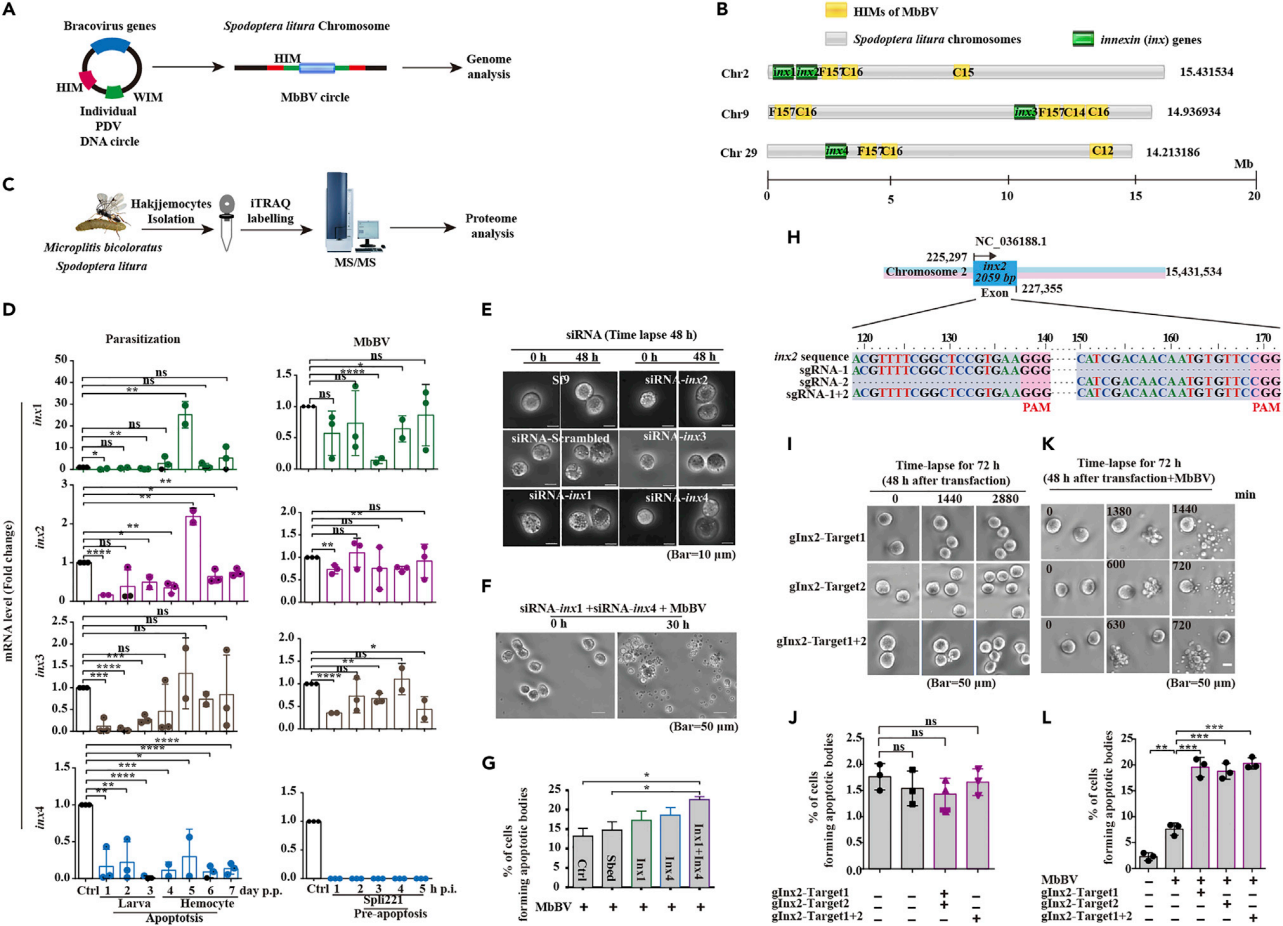
MbBV inhibited innexins (Inxs) (A) Schematic of MbBV integrated into the genome of *Spodoptera litura* at host integration motifs (HIMs). (B) Four *inx* genes in chromosomes of *S. litura* and location of viral DNA integration in genome. (C) Schematic of proteome analysis. (D) qRT-PCR analysis of the expression of four *inx* genes during natural parasitization (p.p., post-parasitization) and infection by MbBV (p.i., post-infection). Haemocytes in which apoptosis was induced by natural parasitism; Spli221 cells in which pre-apoptosis was induced by infection with MbBV particles. ∗p < 0.05, ∗∗p < 0.01, ∗∗∗p < 0.001, ∗∗∗∗p < 0.0001, ns, no significant differences. Unpaired Student's *t*-test with Holm-Sidak method for multiple *t* test; n = 3. (E) Time-lapse microscopy of cells treated with siRNA. Scale bar, 10 μm. (F and G) Time-lapse microscopy of cells treated with siRNA and MbBV. (H) Schematic of gRNA of *inx2*. (I**–**L) CRISPR/Cas9-mediated knockdown of *inx2* in the presence/absence of MbBV infection. Scale bar, 50 μm. The number of cells forming apoptotic bodies. ∗∗p < 0.01, ∗∗∗p < 0.001, ∗∗∗∗p < 0.0001, ns, no significant differences. Unpaired Student's *t*-test with Holm-Sidak method for multiple *t* test; n = 3. See also Figure S1, Tables S1 and S2, and Video S4.

Inxs form hemichannels in invertebrates (Güiza et al., 2018; Luo and Turnbull, 2011). To examine the regulation of Inx proteins by MbBV for hemichannel closure, we performed genome analysis (Figure 2A) of *M. bicoloratus*-parasitized hemocytes of *S. litura* and MbBV-infected Spli221 cell line hemocytes by searching for host integration motifs (HIMs) (Beck et al., 2011; Chevignon et al., 2018) to identify the sites of viral integration into host DNA. Four *inx* genes were found in three chromosomes, 2, 9, and 29, among the 31 pairs of chromosomes in *S. litura* (Cheng et al., 2017). The HIMs, HIM-C16, and HIM-F157 (Figure S1A) were found near *inx1* and *inx2* in chromosome 2, indicating that MbBV DNA was not inserted into *inx1* and *inx2* (Figure 2B). Similarly, HIM-C16, HIM-C14, and HIM-F157 were found near *inx3* in chromosome 9 (Figure 2B), and HIM-C16 and HIM-F157 were found near *inx4* in chromosome 29 (Figure 2B). Therefore, MbBV DNA was not inserted into any of the four *inx* genes, which led us to question whether this location affected *inx* expression (Table S1).

In addition, we performed proteome analysis (Figure 2C) of the hemocytes of *S. litura*. The proteomics data analysis revealed an absence of Inx1 and Inx4 proteins, normal levels of Inx2, and decreased levels of Inx3 (Table S2). To confirm these data, we measured the mRNA levels in hemocytes from *S. litura* larvae after parasitization by the wasp and in Spli221 cells infected by MbBV. *inx1* mRNA could be detected in both the larvae and the hemocytes, albeit inconsistently, and the expression of *inx2* and *inx3* mRNAs was similar, whereas that of *inx4* mRNA was consistently downregulated by MbBV (Figure 2D). The hemocyte expression of *inx1*, *inx2*, and *inx3* was similar in MbBV-infected Spli221 cells; however, no *inx4* mRNA was detected (Figure 2D). Combining the results of the mRNA and protein analyses, we concluded that MbBV downregulated *inx1* and *inx4* expression and inhibited Inx1 and Inx4 synthesis in hemocytes during parasitization and infection by MbBV, whereas *inx2/3* mRNA and protein continued to be expressed.

As these findings led to further questions regarding the roles of Inx1 and Inx4 in apoptotic body formation, we designed siRNAs to knock down the expression of all 4 *inx* genes (Figure S1B). Unexpectedly, none of the siRNAs triggered apoptotic body formation when used alone (Figure 2E, Video S4); however, the treatment of MbBV-infected cells with the combination of siRNAs against *inx1* and *inx4* significantly increased apoptotic body formation (Figures 2F and 2G). To confirm these results, we employed the CRISPR/Cas9 system using *inx2* gRNA (Figure 2H) and found that although no apoptotic bodies were formed when we used gInx2-Target1 and gInx2-Target2, both alone and together (Figures 2I and 2J), apoptotic body formation increased in the presence of both the gRNAs and MbBV infection (Figures 2K and 2L). These results suggest that cooperation between MbBV infection and loss of *inx* is necessary for apoptotic body formation.

Video S4. Inx siRNA did not trigger the formation of apoptotic bodies, related to Figure 2

### MbBV-pI3K/AKT-caspase-3 modulated Inx expression

**Figure 3:**
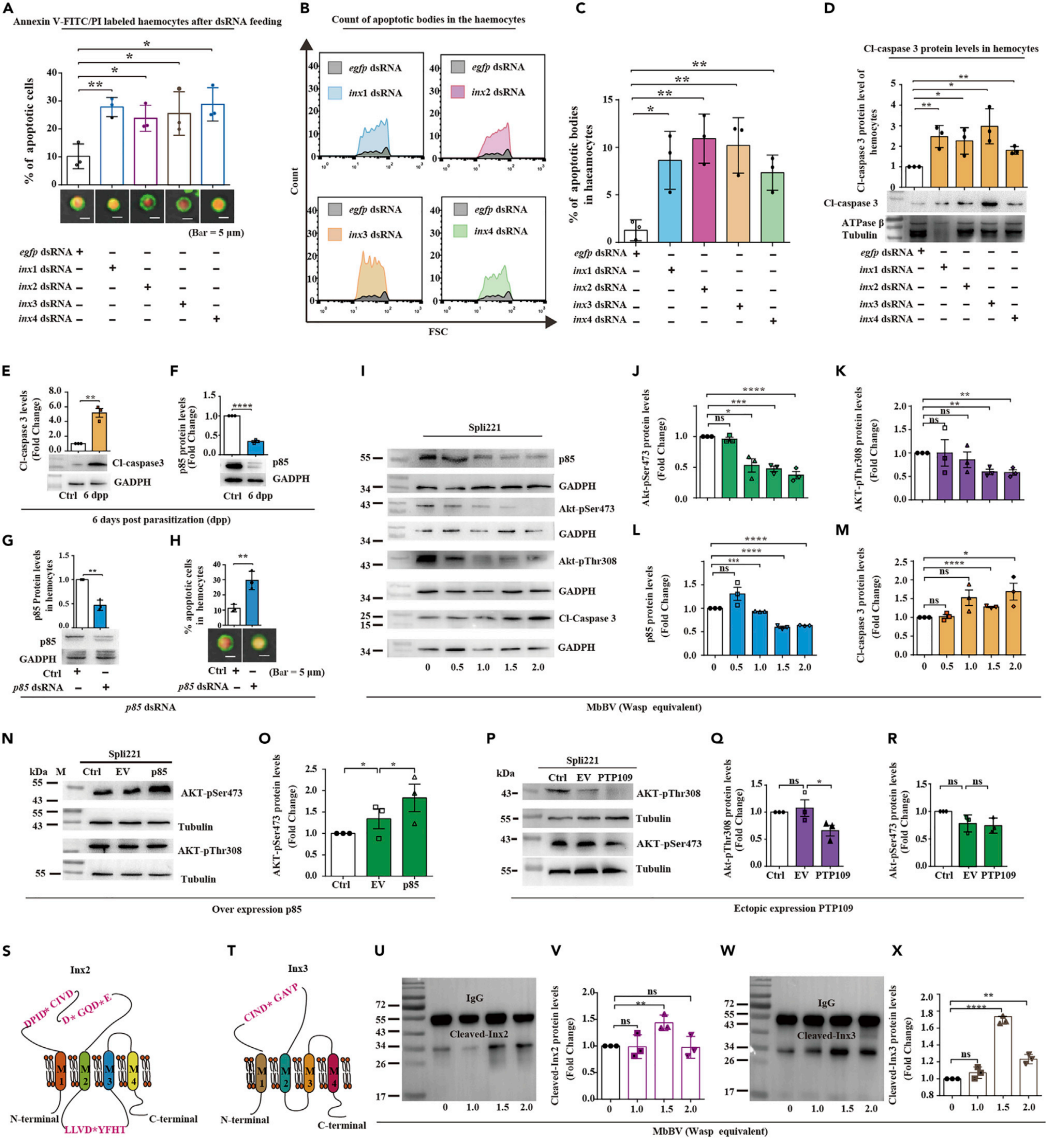
Innexins (Inxs) are substrates of caspase-3 activated by MbBV (A) Annexin V/PI-based detection of apoptotic hemocytes after dsRNA feeding. Scale bar, 5 μm. ∗p < 0.05, ∗∗p < 0.01, unpaired Student's *t*-test with Holm-Sidak method for multiple *t* test; n = 3. (B and C) Flow cytometric detection of apoptotic bodies in the hemocytes after *inx*-dsRNA feeding. The number of apoptotic bodies after dsRNA feeding to the host. ∗p < 0.05, ∗∗p < 0.01, unpaired Student's *t*-test with Holm-Sidak method for multiple *t* test; n = 3. (D) Western blot of cleaved (activated) caspase-3 (Cl-caspase-3) after host feeding with 4 *inx*-dsRNA. ∗p < 0.05, ∗∗p < 0.01, unpaired Student's *t* test with Holm-Sidak method for multiple *t* test; n = 3. ATPase beta chain and tubulin as reference. (E and F) Western blot of Cl-caspase-3 and p85 in hemocytes 6 days post-parasitization (6 dpp). GAPDH was used as reference. (G and H) dsRNA-mediated knockdown of *p85* and apoptotic cell detection. Scale bar, 5 μm. ∗∗p < 0.01, ∗∗∗p < 0.001, ∗∗∗∗p < 0.000; n = 3. (I–M) Western blot of p85, AKT-p-Ser473, AKT-p-Thr308, and Cl-caspase-3 levels with different MbBV doses. GAPDH was used as reference. ∗p < 0.05, ∗∗p < 0.01, ∗∗∗p < 0.001, ∗∗∗∗p < 0.0001, ns, no significant differences. Unpaired Student's *t*-test with Holm-Sidak method for multiple *t* test; n = 3. (N–R) Western blot of AKT-p-Ser473 and AKT-p-Thr308 after *p85* overexpression. ∗p < 0.05, ns, no significant differences. Unpaired Student's *t*-test with Holm-Sidak method for multiple *t* test; n = 3. (S and T) Schematic of caspase-3 cleavage sites. (U–X) Western blot of cleavage of Inx2 and Inx3 by MbBV and activated caspase-3. ∗p < 0.05, ∗∗∗∗p < 0.0001, ns, no significant differences. Unpaired Student's *t*-test with Holm-Sidak method for multiple *t* test; n = 3. See also Figures S2–S4.

To identify the factors responsible for Inx-mediated apoptotic body formation, we generated dsRNAs against all four *inx* genes (Figure S2) by feeding *S. litura* larvae and also determined the levels of apoptosis (Figure 3A) and apoptotic body formation (Figure 3B). We found that all four *inx* dsRNAs increased the number of apoptotic cells (Figure 3A), and flow cytometry analysis (Figure S3) revealed a significant increase in apoptotic body formation (Figures 3B and 3C), which suggests that Inx depletion can trigger apoptosis even in uninfected larvae.

Cleaved caspase-3, which has been found to cleave connexin45.6 (Cx45.6) (Yin et al., 2001), was detected in the hemocytes of larvae after dsRNA administration (Figure 3D). Indeed, cleaved caspase-3 levels were significantly higher (Figure 3E) in hemocytes after parasitization, whereas p85 levels had decreased considerably (Figure 3F). The reduction in p85 levels by treatment with dsRNA directed against the *p85* gene increased the number of apoptotic hemocytes (Figures 3G and 3H). These results led to the investigation of the interaction between the pI3K/AKT signaling pathway, specifically via Ser473 and Thr308 of AKT, and MbBV. We found a viral-dosage-dependent decrease in the levels of AKT-Ser473 phosphorylation (Figures 3I and 3J), AKT-Thr308 phosphorylation (Figure 3K), and p85 (Figure 3L), accompanied by an increased level of cleaved caspase-3 (Figure 3M). To confirm whether p85 phosphorylated AKT-Ser473 and Thr308, we overexpressed *p85* in the Spli221 cell line and found an increase in the levels of AKT-pSer473 but not of AKT-pThr308 (Figures 3N and 3O), suggesting the involvement of an MbBV-mediated decrease in p85-catalyzed phosphorylation of Ser473 in the observed apoptotic effects.

Next, we investigated the factors from MbBV that could dephosphorylate Thr308. As PTP is known to dephosphorylate Thr308 in AKT and the levels of MbBV PTP109 are high during parasitization, we overexpressed *PTP109* in Spli221 cells and found that AKT-Thr308 was dephosphorylated (Figures 3P and 3Q) to a greater extent than Ser473 (Figure 3R). Taken together, these results indicate that activated (cleaved) caspase-3, generated by MbBV, regulated Inx protein levels, which decreased p85-mediated phosphorylation of AKT-Ser473 and, along with viral PTP109-mediated dephosphorylation of AKT-Thr308, led to the inhibition of the pI3K/AKT signaling pathway.

As Cx45.6 is a substrate of caspase-3 (Yin et al., 2001), we examined whether Inx proteins are also substrates of caspase-3. Interestingly, Inx2 and Inx3 have cleavage sites for caspase-3 (Figures 3S and 3T), and immunoprecipitation results revealed that Inx2 and Inx3 were cleaved by activated caspase-3 (Figures S4A and S4B). Immunoprecipitation results revealed that both activated caspase-3 and MbBV infection led to the cleavage of Inx2 and Inx3 in Spli221 cells (Figures S4C and S4D) to ∼34 kDa fragments. The Spli221 cells showed basal levels of cleaved Inx2 and Inx3 (Figures S4E and S4F) and the cleavage of Inx2 and Inx3 followed a vial-dosage-dependent pattern (Figures 3U–3X). These results indicate that Inx proteins are substrates of activated caspase-3, which is generated by MbBV-mediated cleavage, and that the cleavage of Inx proteins decreased the p85-mediated effects and increased the PTP109-mediated effects on AKT-Ser473 and AKT-Thr308 phosphorylation, respectively.

### Hemichannel opening reduced apoptosis

**Figure 4:**
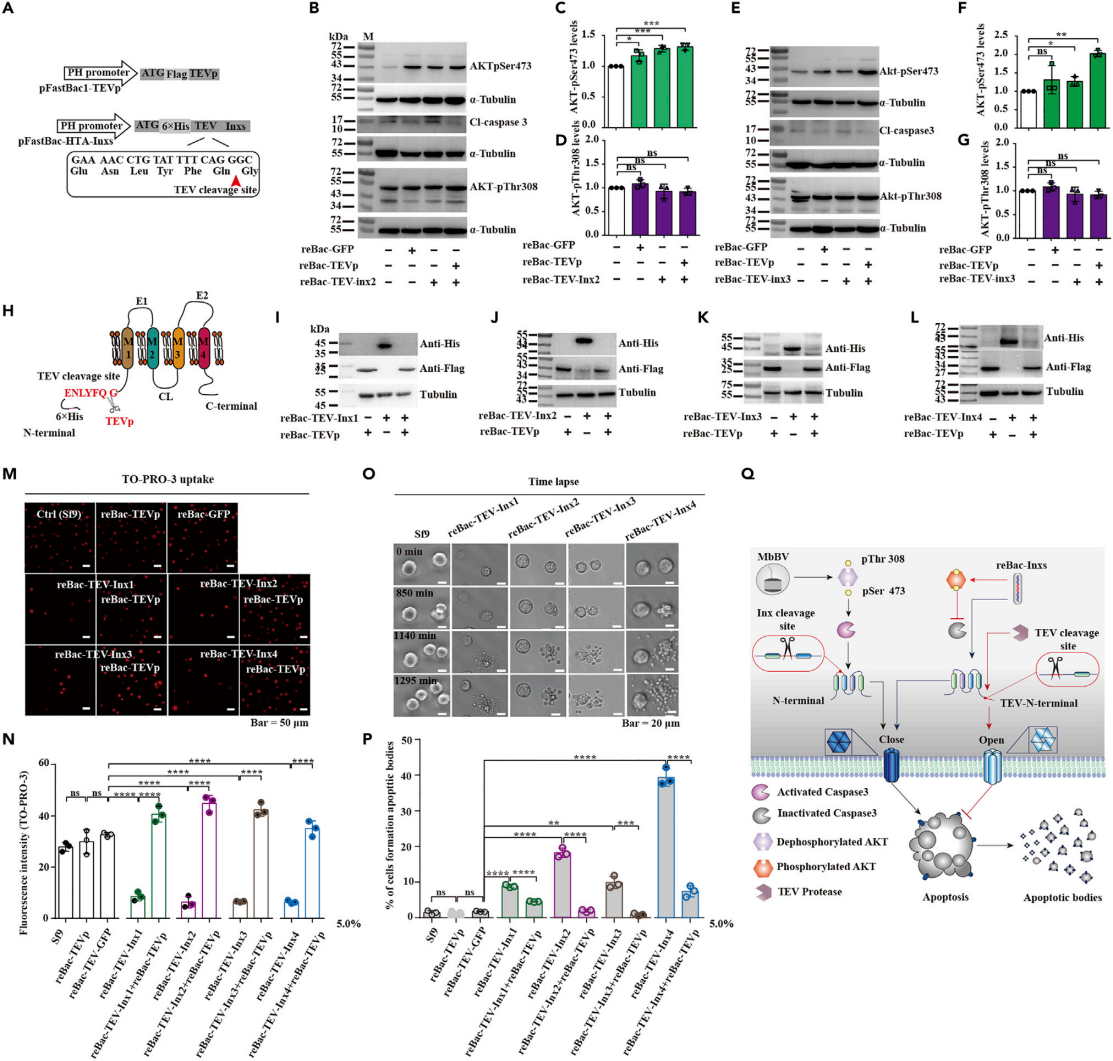
Reduction in apoptotic body formation upon opening of innexin hemichannel (A) Schematic of vectors of reBac-TEV-Inxs and reBac-TEV protease (re-Bac-TEVp). (B–D) Western blot of AKT-pSer473, AKT-pThr308, and Cl-caspase-3 in cells transduced with reBac-TEV-Inx2 and/or reBac-TEVp. ∗p < 0.05, ∗∗p < 0.01, ∗∗∗p < 0.001, ∗∗∗∗p < 0.0001, ns, no significant differences. Unpaired Student's *t*-test with Holm-Sidak method for multiple *t* test; n = 3. (E–G) Western blot of AKT-pSer473, AKT-pThr308, and Cl-caspase-3 in cells transduced with reBac-TEV-Inx3 and/or reBac-TEVp cells. ∗p < 0.05, ∗∗p < 0.01, ∗∗∗p < 0.001, ∗∗∗∗p < 0.0001, ns, no significant differences. Unpaired Student's *t*-test with Holm-Sidak method for multiple *t* test; n = 3. (H) Schematic of TEV cleavage sites in reBac-TEV-Inxs and reBac-TEVp. (I–L) Western blots of Inxs after co-infection with both reBac-TEV-Inxs and reBac-TEVp. (M and N) Dye uptake through open hemichannels formed by Inxs after cleavage by TEVp. (O and P) Time-lapse microscopy of apoptotic bodies after cleavage of Inxs by TEVp; scale bar, 50 μm. (Q) Schematic of the mechanism of Inx dysregulation by MbBV for the formation of apoptotic bodies via the activation of caspase-3. MbBV promotes pThr308 and pSer473 dephosphorylation to activate caspase-3, which cleaves Inx2 and Inx3, causing apoptotic cell hemichannel closure and forming apoptotic bodies. In contrast, TEV-N-terminal cleavage sites containing reBac-Inxs promote pThr308 and pSer473 phosphorylation to inhibit caspase-3; TEV proteases cleave N-terminal sites to recover Inxs, open hemichannels, and inhibit cell apoptosis.

Based on the above results, we sought to confirm our conclusion that MbBV closed hemichannels by activating caspase-3 to trigger apoptotic cell disassembly. We used reBac-TEV-Inx2 and reBac-TEV-Inx3, which had shown hemichannel closure in infected cells (Chen et al., 2016; Guo et al., 2015), and used a tobacco etch virus (TEV) protease to cleave the TEV sites of the two reBac-TEV-Inxs (Figure 4A). Specifically, cells infected by reBac-TEV-Inxs showed an increase in AKT-pSer473 levels, highly stable AKT-pThr308 levels, and reduced cleavage of caspase-3; resultantly, Inx cleavage was limited (Figures 4B‒4G).

Next, in the process of Inx recovery, cell co-infection with reBac-TEV-Inxs and reBac-TEVp resulted in the cleavage of the TEV sites and the loss of a 6×His fragment from the N-terminal ends of the Inx proteins (Figure 4H). All four recovered Inx proteins were detected when the cells were co-infected with both reBac-TEV-Inxs and reBac-TEVp (Figures 4I‒4L). Notably, the recovered Inx proteins led to the opening of the Inx hemichannels (Figures 4M and 4N). Simultaneously, the number of apoptotic cells was confirmed to decrease significantly (Figures 4O and 4P), suggesting that the opening of the Inx hemichannels reduced cell disassembly.

Taken together, our results show that MbBV dephosphorylated AKT and activated caspase-3, which cleaved the Inx proteins, closed hemichannels, and promoted apoptotic cell disassembly; additionally, opening of the closed hemichannels reduced the formation of apoptotic bodies (Figure 4Q).

## Discussion

The results of this study enable the advancement of several concepts. First, we identified that MbBV induced hemichannel closure to trigger apoptosis and promote apoptotic cell disassembly. Second, the cleavage of Inxs by activated caspase-3 was responsible for hemichannel closure mediated by MbBV via the suppression of pI3K/AKT signaling. Third, the opening of hemichannels formed by Inxs suppressed apoptosis via the modulation of Inx levels and inhibition of cell-cell communication, which attenuated immunosuppression in invertebrates and vertebrates.

Contrary to the view that the opening of hemichannels mediates apoptosis (Chandrasekhar and Bera, 2012; Hur et al., 2003), our study showed that MbBV promotes the unconventional apoptosis pathway of infected cells. We propose that, in the invertebrate host, the hemichannel switching mechanism is related to cell disintegration, which is consistent with the finding that pannexin1 channel activity is negatively correlated with the number of apoptotic bodies (Poon et al., 2014).

It is now well known that connexin (Yin et al., 2001) and pannexin (Ruan et al., 2020) are regulated by caspase, and our results also show that Inxs are regulated by caspase-3, completing the mechanism of interaction between the connexin and caspase families. Meanwhile, we also found that MbBV relies on pI3K/AKT signaling to release caspase to induce and accelerate cell apoptosis; this result is different from viruses that also use pI3K/AKT and need to replicate. For example, the enterovirus EV71 activates AKT to inhibit cell apoptosis in the early stages of infection and inhibits AKT phosphorylation to promote cell apoptosis until the late stage of infection (Zhang et al., 2015). This suggests that MbBV may have a different infection mechanism from ordinary viruses and needs to be explored further.

The intercellular transmission of small molecules plays a key role in the regulation of cell tissue homeostasis (Chen et al., 2021). For the immune system, cellular communication mediated by small molecules is particularly important due to the lack of gap junctions. Panx1 has been proven to be widely present in mammalian macrophages (Marina-Garcia et al., 2008), neutrophils (Chen et al., 2010), T cells (Orellana et al., 2013), B cells, and dendritic cells (Saez et al., 2014). Some studies have reported that the main function of the Panx channel is to release ATP (Pelegrin and Surprenant, 2006, 2007). Extracellular ATP is closely involved in the immune response and is usually a pro-inflammatory factor (Faas et al., 2017); however, it may also have anti-inflammatory properties under certain conditions, and its role in the immune response depends on the relative balance between its inflammatory properties (Faas et al., 2017). Therefore, hemichannel closure blocks the transmission of immune signals and inhibits the immune response.

In conclusion, this study revealed a mechanism whereby MbBV-mediated hemichannel closure was activated in MbBV infection-induced immunosuppression during the parasitization of *S. litura* via the inhibition of pI3K/AKT signaling; additionally, apoptosis was also promoted by the activation of caspase-3, a manifestation of the “hemichannel open and close” theory of regulated cellular immune response.

### Limitations of the study

In terms of the limitations, our data are primarily based on the Microplitis bicoloratus bracovirus (MbBV)-*Microplitis bicoloratus*-*Spodoptera litura* model. Although we confirmed MbBV cellular immunity via hemichannel closure in innate immunity, the regulation of humoral immunity by MbBV may affect hemichannels. Given that the novel “hemichannel opening and closure” model proposes the global regulation between cellular immunity and humoral immunity, further investigation will be necessary to fully understand the molecular mechanisms underlying the link between cellular immunity-hemichannels-humoral immunity in innate immunity during MbBV infection.

### Resource availability

#### Lead contact

Further information and requests for resources and reagent should be directed to and will be fulfilled by the lead contact, Kai-Jun Luo (kaijun_luo@ynu.edu.cn).

#### Material availability

This study did not generate new unique reagents.

#### Data and code availability

This study did not generate data sets.

## Methods

All methods can be found in the accompanying transparent methods supplemental file.

## Supplemental information

Document S1. Transparent methods, Figures S1–S4, and Tables S1 and S2
