# Supplementary material for: Bracovirus-mediated innexin hemichannel closure in cell disassembly
Source: iScience. 2021 Mar 8;24(4):102281. doi: 10.1016/j.isci.2021.102281 (PMC8008186; doi:10.1016/j.isci.2021.102281)
Supplement: Document S1. Transparent methods, Figures S1–S4, and Tables S1 and S2 [file mmc1.pdf]

iScience, Volume *24*

Supplemental information

Bracovirus-mediated innexin hemichannel closure in cell disassembly

Chang-Xu Chen, Hao-Juan He, Qiu-Chen Cai, Wei Zhang, Tian-Chao Kou, Xue-Wen Zhang, Shan You, Ya-Bin Chen, Tian Liu, Wei Xiao, Qi-Shun Zhu, and Kai-Jun Luo

iScience

Supplemental Information

Bracovirus-mediated innexin-hemichannel closure in cell disassembly

Chang-Xue Chen, Hao-Juan He, Qiu-Chen Cai, Wei Zhang, Tian-Chao Kou, Xue-Wen Zhang, Shan You, Ya-Bin Chen, Tian Liu, Wei Xiao, Qi-Shun Zhu, Kai-Jun Luo

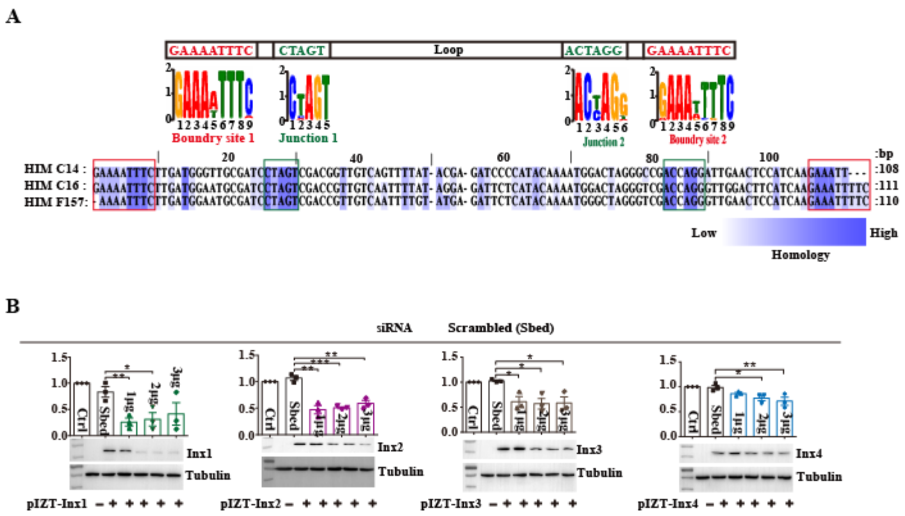

Figure S1. Host integration motifs (HIMs) and siRNA (Related to Figure 2)

(A) HIM in C14, C16, and F157. (B) Levels of the four Inx proteins after siRNA-dose-dependent knockdown.

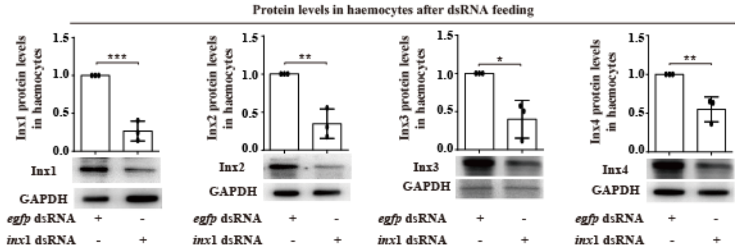

Figure S2. dsRNA inhibited Inx proteins (Related to Figure 3).

Western blotting of four Inx proteins from haemocytes isolated from host treated with dsRNAi. ds*egfp* was used as a dsRNA control and GAPDH as a reference.

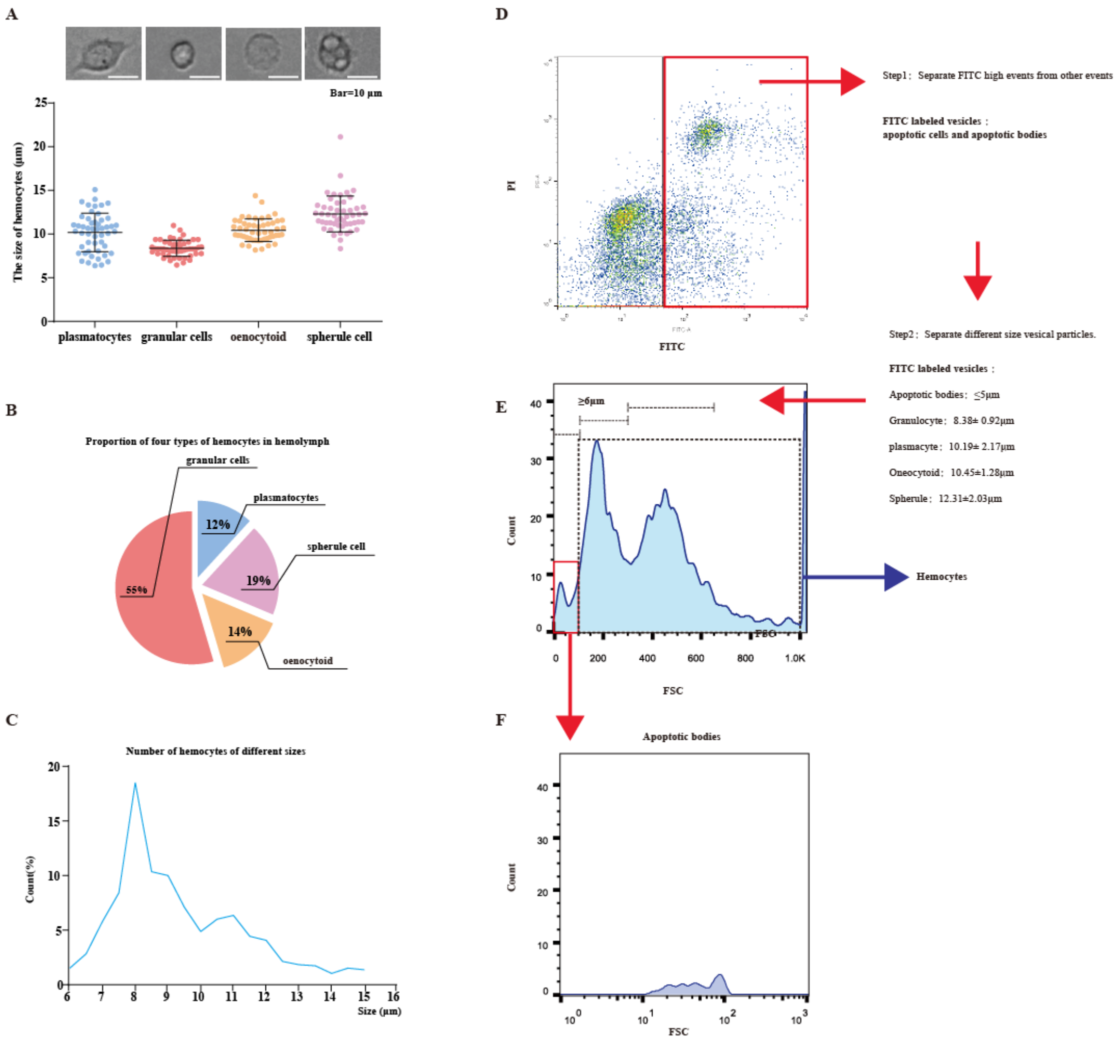

Figure S3. Flow cytometry analysis of the number of apoptotic bodies (Related to Figure 3).

(A) Size distribution of haemocytes of *Spodoptera litura*: Granulocyte, 8.38 ± 0.92 μm (n = 50); plasmacyte, 10.19 ± 2.17 μm (n = 50); Oneocytoid: 10.45 ± 1.28 μm (n = 50); Spherule, 12.31 ± 2.03 μm (n = 50). (B) Proportion of different blood cells in the haemolymph of *S. litura*. (C) Percentage of haemocytes of different sizes in *S. litura*. Different types of haemocytes were analysed according to their size to obtain a cell size ratio map with two peaks. (D) Screening of apoptotic cell populations of haemocytes of *S. litura* using flow cytometry. (E) Apoptotic cell population containing apoptotic bodies and apoptotic cells, identified by peak and FSC, compared with the line graph of the percentage of cell size calculated by microscopy. The apoptotic body population is indicated by the smallest peak. (F) Number of apoptotic bodies.

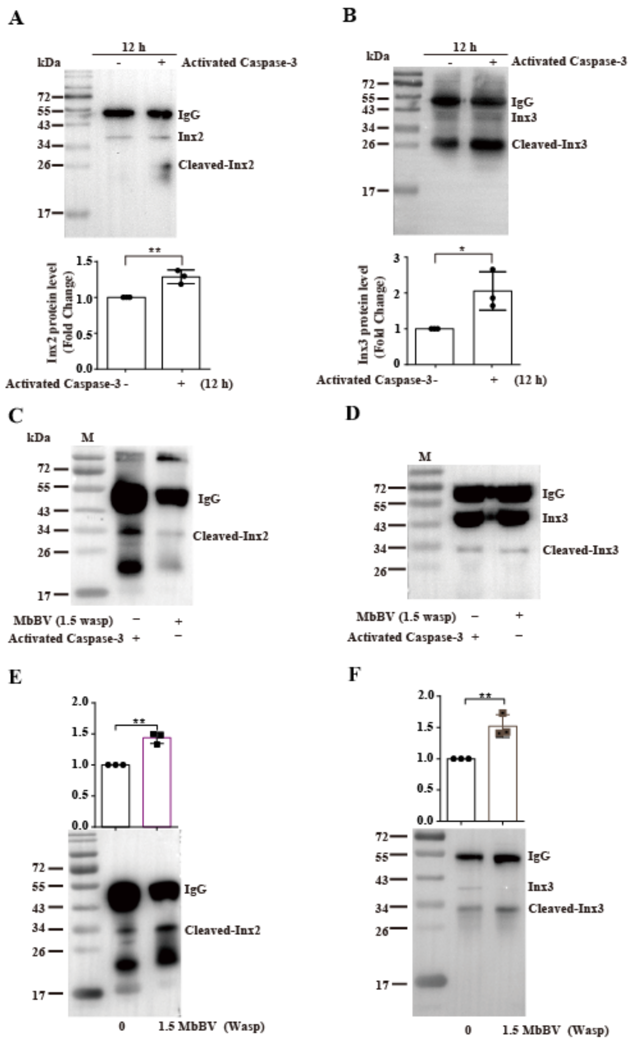

Figure S4. Innexins (Inxs) are substrates of caspase-3 activated by MbBV (Related to Figure 3).

(A and B) Western blot of Inx2/3 cleaved by active caspase-3. (C and D) Western blot of Inx2/3 cleaved by active caspase-3 and MbBV. (E and F) Western blot of Inx2/3 cleaved by MbBV. **$p < 0.01$. Unpaired Student's *t*-test using the Holm-Sidak method for multiple *t*-test; n = 3. IgG was used as a reference.

Table S1. The location of Inx genes in bracovirus integrated *Spodoptera litura* genome (Related to Figure 2).

ID	Name	CDS length (bp)	Position	MbBV DNA integration nearby
SWUS10010590	*inx1*	1086	Chr 2	HIM-C16, HIM-F157
SWUS10010610	*inx2*	1080	Chr 2	HIM-C16, HIM-F157
SWUS10043510	*inx3*	1161	Chr 9	HIM-C16, HIM-C14, HIM-F157
SWUS10127310	*inx4*	1116	Chr 29	HIM-C16, HIM-F157

Table S2. Inx proteins in *Spodoptera litura* haemocytes parasitised by *Microplitis bicoloratus* (Related to Figure 2).

Accession	Name	M/S results	Protein Mass	Protein Length	% Cov (95)	Unique Peptides	Unique Peptide Sequence
comp88846_c0_seq1	Inx1	/	/	/	/	/	/
comp65035_c0_seq1	Inx2	/	41526.3	360	8.635000139	2	MLVLDLNCPVVGDECKDSR; LAPQAQVEAVAR
Comp99381_c0_seq1	Inx3	down	43965.8	387	15.02999961	5	GIAHPGLGNDFEEEKR; LVQYLVDTR; TDPMIEVFPR; VFGEVLDELSR; FGTPAGVESLVR
Comp121018_c0_seq1	Inx4	/	/	/	/		/

Transparent Methods

Reagents. TO-PRO-3 was purchased from Invitrogen (Thermo Fisher, T3605, Eugene, OR， America). Annexin V-FITC/PI was purchased from Solarbio (Solarbio, CA1020, Beijing, China). PI was purchased from Sigma. Activated caspase-3 was purchased from Cayman (Michigan, 10010209), and CBX was purchased from Sigma.

Microscopy. Time-lapse live imaging was performed at 27 °C using a Leica DMi8LASX microscope with a 20× objective lens.

Insect rearing and virion isolation. To identify the apoptotic bodies of haemocytes parasitised by *Microplitis bicoloratus*, *Spodoptera litura* was reared as described previously (Luo and Pang, 2006; Luo et al., 2007).

Cell culture. Sf9 (IPLB-Sf21-AE) cells were derived from *S. frugiperda* pupal ovarian tissue (Vaughn et al., 1977), and adherent Spli221 (TUAT-Spli221) cells were derived from *S. litura* (Yanase et al., 1998) cultured in TNM-FH insect culture medium supplemented with 10% foetal bovine serum (Hyclone, Logan, UT, USA).

Apoptotic bodies. To identify the apoptotic bodies of haemocytes, 100 μL of a suspension of haemocytes isolated from *S. litura* larvae was mixed with 500 μL of chilled 1× PBS and dispensed in 12-well plates, followed by incubation for 20 min. Once all haemocytes adhered to the bottom of the plate, the cells were labelled using

an Annexin/PI kit purchased form Solarbio (CA1020, Beijing, China). Five images per recorded per 12-well plate using the 20× objective lens (both bright and fluorescent fields) of an inverted fluorescence microscope (Olympus 1X71) at room temperature. Vesicles < 5 μm in size were defined as apoptotic bodies. The total number of cells in a single image was counted; five images were acquired per experiment.

Time-lapse microscopy was used to analyse apoptotic body formation during different treatments with MbBV and reBac-TEV-Inxs. After 2 h of incubation of 1 × 10^6 cells/well in 6-well plates (Nest, 703001), MbBV was added to the plates, and five images were recorded per plate using the 20× objective lens (both bright and fluorescent fields) at 30-min intervals for 72 h using a live-cell imaging system (Leica, DMi8LASX). LAS X software was used to analyse and record the number of cells forming apoptotic bodies. The percentage of apoptotic bodies = (apoptotic bodies/total cell number)/100.

Flow cytometry gating for apoptotic bodies. The *S. litura* haemocytes were stained with Annexin V-FITC/PI. Flow cytometry analysis helped sort a subpopulation of cells with high FITC staining, which included apoptotic cells and apoptotic bodies. Next, the high FITC cell subpopulation was analysed using FSC and Count as the horizontal and vertical coordinates, to detect three peaks within the cell population. Comparison of the haemocyte data using microscopy confirmed that the FSC value was within the expected range of 100-1.0 k, similar to the distribution of a normal haemocyte population. The FSC values under the first and second count peak should be

approximately equal to 8 and 11 μm, respectively, whereas the FSC value corresponding to the first count value is approximately equal to 6 μm. In addition, each particle group with a complete membrane structure with a size ≤ 5 μm was identified as an apoptotic body group.

Induction of apoptosis. For *in vivo* experiments, the haemocytes of *S. litura* were parasitised by *M. bicoloratus*. For *in vitro* experiments, Sf9 cells in TNM-FH supplemented with 10% FBS were treated with 1–9 wasp equivalents of bracovirus or 1%–5% reBac-TEV-Inxs for 12–72 h.

Dye uptake via hemichannels. To measure dye uptake by apoptotic cells treated with viruses, the cells were incubated with TO-PRO-3 (Thermo Fisher, T3605，Eugene, OR，America) for 15 min at room temperature. Five images were recorded per plate using the 20× objective lens (both bright and fluorescent fields) of an inverted fluorescence microscope (Olympus 1X71) at room temperature. PI uptake by apoptotic cells treated with MbBV was measured at 4 °C, as described previously (Luo and Turnbull, 2011). Briefly, 1 × 10^4 cells, treated as indicated, were seeded in 96-well plates and incubated at 4 °C for 2 h. This was followed by incubation with 50 μg/mL of PI for 5 min at 4 °C. The cells were fixed for 15 min with 3.7% formaldehyde, and five images were recorded per plate using the 20× objective lens (both bright and fluorescent fields) of an inverted fluorescence microscope (Olympus 1X71, Tokyo, Japan) at room temperature.

Genome analysis of MbBV-infected cells. To scan for fragments of MbBV integrated into the host genome, we sequenced DNA isolated from 6-day parasitised haemocytes and MbBV-infected Spli221 cells. Host integration motifs (HIMs) from Microplitis demolitor bracovirus (Burke et al., 2014) were used to scan chromosomes 2, 9, and 29, where Inx1, Inx2, Inx3, and Inx4 are localised. The MbBV genome sequence was compared with these sequences.

Proteomics of parasitised haemocytes. To examine the expression of *inx* genes after parasitisation, the parasitised haemocytes were isolated, and protein sequences for Inx 1–4 were determined using tandem mass spectrometry.

qRT-PCR. Total RNA was isolated from five samples using RNAiso Plus (TaKaRa, Dalian, China), according to the manufacturer's instructions, followed by DNase treatment. The concentration and purity of each RNA sample were determined by measuring the optical density ratio A260/A280 using a NanoDrop 2000. Samples with an A260/A280 ratio > 2.0 were used to synthesise cDNA using a 5× All-In-One RT MasterMix Kit (abm, Vancouver, Canada) according to the manufacturer's instructions. All cDNA samples were stored at −80 °C for preservation. qRT-PCR was performed using cDNA and the following primers: Q-Inx1-F (5'- GCG GTA GAG CGG ACA C -3'), and Q-Inx1-R (5'- CGT GAT GCG AGG GAA TA -3'); Q-Inx2-F (5'-CGT TCC GTT TCT TTA TCT G-3'), and Q-Inx2-R (5'- ACA CGC TCC TCT GGC TC-3'); Q-

Inx3-F (5’-ATC GCA TCA CAT CAG CC-3’), and Q-Inx3-R (5’-AGG TAA TCC AGC AAT AGG-3’); Q-Inx4-F (5’- AAG ACG CCA TCA ACA GC -3’); Q-Inx1-R (5’- GCC GAG CAG CAC AAA -3’); Q-18S-F (5’-AGA ACT CTG ACC AGT GAT GGG ATG-3’), Q-18S-R (5’-CTG ATT CCC CGT TAC CCG TGA-3’). We used EvaGreen 2× qPCR MasterMix (Abm, MasterMix , Richmond, Canada) with the following recommended cycling parameters: 95 °C, 30 s; 95 °C, 5 s, 60 °C, 34 s, 40 cycles; 95 °C, 15 s; 60 °C, 1 min; 95 °C, 15 s. The 18S rDNA gene was used as the reference gene. To quantify the relative mRNA levels of each target gene, each sample was tested in triplicate, and the $2^{-\Delta\Delta CT}$ method was used as previously described (Livak and Schmittgen, 2001).

Gene knockdown. siRNAs were used to knock down the genes of interest. The siRNAs were synthesised by GenePharma (GenePharma, Suzhou, China). The sequences were as follows: siRNA-*inx*1-F (5’-GGA CUG AUA AUG CAG UGU UTT-3’), and siRNA-*inx*1-R (5’-AAC ACU GCA UUA UCA GUC CTT-3’); siRNA-*inx*2-F (5’-GGU GAA AUA CCA CAA GUA UTT-3’), and siRNA-*inx*2-R (5’-AUA CUU GUG GUA UUU CAC CTT-3’); siRNA-*inx*3-F (5’-GGA GGU GCU UUC UUG ACA UTT-3’), and siRNA-*inx*3-R (5’-AUG UCA AGA AAG CAC CUC CTT-3’); siRNA-*inx*4-F (5’-GCG AGA AGG ACA GUG AUA ATT-3’), and siRNA-*inx*4-R (5’-UUA UCA CUG UCC UUC UCG CTT-3’). CRISPR/Cas9 was used to knock out the gene of interest; gRNAs were designed using the resource available at http://sidirect2.rnai.jp. To knock down gene expression *in vivo*, dsRNA plasmids were constructed as described

previously (Timmons et al., 2001). Briefly, the gene sequences were clone into an L4440RNAi vector containing two convergent T7 polymerase promoters that were oppositely oriented and separated by a multicloning site. The plasmids were sequenced, and the correctly cloned plasmids were used to transform *Escherichia coli* HT115 (DE3), which was the bacterial host. Bacteria transformed with EGFP RNAi served as negative controls.

Western blotting. Western blotting was performed as previously described (Liu et al., 2013). Briefly, the cultured cells were lysed using RIPA lysis buffer (cat. no. R0100; Solarbio, Beijing, China), and the protein concentrations were measured using a BCA protein quantification assay kit (cat. no. BCA02; Dingguo, Beijing, China). Samples (50 µg) were separated by SDS-polyacrylamide gel electrophoresis and transferred to PVDF membranes. After incubation with antibodies, the bands were visualised using enhanced chemiluminescence (Beyotime).

Caspase-3-mediated cleavage of Inxs. Inx proteins were isolated by incubating anti-Inx2 and anti-Inx3 antibodies with cell lysates, followed by incubation with protein A+G agarose beads (Beyotime, cat no. P2012) according to the manufacturer's protocol. The isolated Inx proteins were incubated with active caspase-3 peptide (C10010209, Cayman) at 37 °C and subjected to western blotting after 12–16 h.

TEV-Inxs and TEV protease. reBac-TEV-Inxs and reBac-Flag-TEV protease (N-terminally elongated bacmids) were constructed based on previously described reports

(Chen et al., 2016; Guo et al., 2015). Briefly, pFastBacTM HTA vector (Invitrogen, Carlsbad, CA, USA), which contained a Tobacco Etch Virus (TEV) cleavage site, was used to construct reBac-TEV-Inxs. pFastBac1 containing a Flag tag was used to generate pFasBac1-TEV protease. The plasmids were used to transform competent *E. coli* DH10Bac cells (Gibco), and positive colonies were selected according to the manufacturer's protocol. The same methods were used to generate reBac-TEV-Inx1 and Inx4.

Determining the volumes of high-titer bac-to-bac virus by cell cycle arrest

Recombinant viruses were generated, and the volume of each high-titre virus was determined using cell cycle arrest (Boukarabila et al., 2009). In brief, for the production of the P1 viral supernatant, Sf9 cells in the mid-log phase growth were transfected with the bacmid. At 72 h after transfection, the supernatant (P1 virus) was isolated. For the production of the P2 viral supernatant, 10 mL of the Sf9 cell culture (1.0×10^6 cells/mL) was added to a cell culture flask; 30 min after cell adhesion, 5% (500 μL) P1 virus was added into the flask (If the cells stopped doubling at 24 h, we deduced that excess P1 virus had been added. The process was then repeated using 1% virus. In contrast, if the cells did not stop dividing by 48 h, the procedure was repeated using 10% P1 viral supernatant). At 72 h after the P1 virus infection, the cells were centrifuged at $500 \times g$ for 5 min and the supernatant (P2 virus) was recovered. For preparing the P3 viral supernatant, a large volume of mid-log phase cells (10 mL, 1.0×10^6 cells/mL) were infected with 0.1% (10 μL) P2 virus supernatant. The cells were counted every 24 h for 72 h (if the cells stopped doubling at 24 h, we deduced that excess P1 virus had been added, and the process was then repeated using 0.05% virus. In contrast, if the cells did not stop dividing by 48 h, the process was repeated using 0.5% P2 virus). After 72 h, the cell suspension was centrifuged at $500 \times g$ for 5 min to recover the supernatant (P3 virus). To induce cell apoptosis, 1% of the P3 virus supernatant was used, and 5% of the P3 virus supernatant (MOI of 5% P3 virus $\approx$ 1) was used to induce the formation of apoptotic bodies.

Statistical analyses. Data were analysed using GraphPad Prism (ver. 7, Prism), and statistical significance was determined using the Student's *t*-test for unpaired experiments (two-tailed). $p < 0.05$ was considered to indicate statistically significant difference between groups. The resulting data are presented as means ± SEM from at least three independent experiments.

Supplemental References

Boukarabila, H., Saurin, A.J., Batsche, E., Mossadegh, N., van Lohuizen, M., Otte, A.P., Pradel, J., Muchardt, C., Sieweke, M., and Duprez, E. (2009). The PRC1 Polycomb group complex interacts with PLZF/RARA to mediate leukemic transformation. Genes Dev. *23*, 1195-1206.

Burke, G.R., Walden, K.K.O., Whitfield, J.B., Robertson, H.M., and Strand, M.R. (2014). Widespread genome reorganization of an obligate virus mutualist. PLoS Genet. *10*, e1004660.

Chen, Y.-B., Xiao, W., Li, M., Zhang, Y., Yang, Y., Hu, J.-S., and Luo, K.-J. (2016). N-terminally elongated SpliInx2 and SpliInx3 reduce baculovirus-triggered apoptosis via hemichannel closure. Arch. Insect Biochem. Physiol. *92*, 24-37.

Guo, L.E., Zhang, J.F., Liu, X.Y., Zhang, L.M., Zhang, H.L., Chen, J.H., Xie, X.G., Zhou, Y., Luo, K.-J., and Yoon, J. (2015). Phosphate ion targeted colorimetric and fluorescent probe and its use to monitor endogeneous phosphate ion in a hemichannel-closed cell. Anal. Chem. *87*, 1196-1201.

Livak, K.J., and Schmittgen, T.D. (2001). Analysis of relative gene expression data using real-time quantitative PCR and the $2^{-\Delta\Delta CT}$ method. Methods *25*, 402-408.

Luo, K., and Turnbull, M.W. (2011). Characterization of nonjunctional hemichannels in caterpillar cells. J. Insect Sci. *11*, 6.

Luo, K.-J., and Pang, Y. (2006). Spodoptera litura multicapsid nucleopolyhedrovirus inhibits Microplitis bicoloratus polydnavirus-induced host granulocytes apoptosis. J. Insect Physiol. *52*, 795-806.

Luo, K.-J., Trumble, J.T., and Pang, Y. (2007). Development of *Microplitis bicoloratus* on *Spodoptera litura* and implications for biological control. BioControl *52*, 309-321.

Timmons, L., Court, D.L., and Fire, A. (2001). Ingestion of bacterially expressed dsRNAs can produce specific and potent genetic interference in *Caenorhabditis elegans*. Gene *263*, 103-112.

Vaughn, J.L., Goodwin, R.H., Tompkins, G.J., and McCawley, P. (1977). The establishment of two cell lines from the insect *Spodoptera frugiperda* (Lepidoptera; Noctuidae). In Vitro *13*, 213-217.

Yanase, T., Yasunaga, C., and Kawarabata, T. (1998). Replication of *Spodoptera exigua* nucleopolyhedrovirus in permissive and non-permissive lepidopteran cell lines. Acta Virol. *42*, 293-298.

394
